# Phase II study of cetuximab in combination with cisplatin and docetaxel in patients with untreated advanced gastric or gastro-oesophageal junction adenocarcinoma (DOCETUX study)

**DOI:** 10.1038/sj.bjc.6605319

**Published:** 2009-09-22

**Authors:** C Pinto, F Di Fabio, C Barone, S Siena, A Falcone, S Cascinu, F L Rojas Llimpe, G Stella, G Schinzari, S Artale, V Mutri, S Giaquinta, L Giannetta, A Bardelli, A A Martoni

**Affiliations:** 1 Department of Medical Oncology, S. Orsola-Malpighi Hospital, Bologna, Italy; 2 Department of Medical Oncology, Gemelli Hospital, Rome, Italy; 3 Department of Medical Oncology, Falck Division, Niguarda Cà Granda Hospital, Milan, Italy; 4 Department of Medical Oncology, University of Pisa, Pisa, Italy; 5 Department of Medical Oncology, University of Marche, Ancona, Italy; 6 Laboratory of Molecular Genetics, Institute for Cancer Research and Treatment, University of Turin, Candiolo, Italy

**Keywords:** advanced gastric cancer, cetuximab, cisplatin/docetaxel

## Abstract

**Background:**

The conventional treatment options for advanced gastric patients remain unsatisfactory in terms of response rate, response duration, toxicity, and overall survival benefit. The purpose of this phase II study was to evaluate the activity and safety of cetuximab combined with cisplatin and docetaxel as a first-line treatment for advanced gastric or gastro-oesophageal junction adenocarcinoma.

**Methods:**

Untreated patients with histologically confirmed advanced gastric or gastro-oesophageal adenocarcinoma received cetuximab at an initial dose of 400 mg m^−2^ i.v. followed by weekly doses of 250 mg m^−2^, cisplatin 75 mg m^−2^ i.v. on day 1, docetaxel 75 mg m^−2^ i.v. on day 1, every 3 weeks, for a maximum of 6 cycles, and then cetuximab maintenance treatment was allowed in patients with a complete response, partial response, or stable disease.

**Results:**

Seventy-two patients (stomach 81.9% and gastro-oesophageal junction 18.1%; locally advanced disease 4.2%; and metastatic disease 95.8%) were enrolled. The ORR was 41.2% (95% CI, 29.5–52.9). Median time to progression was 5 months (95% CI, 3.7–5.4). Median survival time was 9 months (95% CI, 7–11). The most frequent grades 3–4 toxicity was neutropenia (44.4%). No toxic death was observed.

**Conclusions:**

The addition of cetuximab to the cisplatin/docetaxel regimen improved the ORR of the cisplatin/docetaxel doublet in the first-line treatment of advanced gastric and gastro-oesophageal junction adenocarcinoma, but this combination did not improve the TTP and OS. The toxicity of cisplatin/docetaxel chemotherapy was not affected by the addition of cetuximab.

Gastric cancer is a considerable health problem worldwide and as such remains a major challenge for the whole oncology community. For the year 2007, gastric cancer was estimated to be the second leading cause of cancer death among men and the fourth among women, with an expectancy of 800 000 cancer-related deaths overall (Parkin *et al*, 2005). There is remarkable geographic variation, with 60% of cases arising in Eastern Asia (Kelley and Duggan, 2003; Parkin *et al*, 2005; Garcia *et al*, 2007). In the European Union, for the year 2002, the number of deaths from gastric cancer was 40 759 among men and 27 980 among women (Bosetti *et al*, 2008). In the same year, in Italy, stomach cancers accounted for 6.7% of cancer deaths in both sexes with 6266 deaths in men and 4686 in women (Malvezzi *et al*, 2008). Although the incidence of gastric cancer is declining in the Western world, adenocarcinomas of the gastro-oesophageal junction (GEJ) are increasing in number (Kelley and Duggan, 2003; Parkin *et al*, 2005; Garcia *et al*, 2007; Bosetti *et al*, 2008; Malvezzi *et al*, 2008).

In patients with resectable cancer, surgery and perioperative therapy are potentially curative treatments. However, the majority of patients with gastric cancer had stage III or IV disease at presentation and thus are candidates for palliative chemotherapy. In advanced disease, the median survival in patients not receiving chemotherapy is 3–4 months. A systematic meta-analysis of randomised studies found a statistically significant advantage of chemotherapy compared with the best supportive care and combination of three-drug regimens containing 5-fluorouracil, anthracyclines, and cisplatin (Wagner *et al*, 2006). New triplet and doublet combinations incorporating docetaxel, oxaliplatin, irinotecan, capecitabine, and S-1 have been evaluated in randomised trials. Although little progress has been made in these trials towards improving the median overall survival time beyond 9 months, the outcome of advanced disease is still disappointing (Van Cutsem *et al*, 2006; Cunningham *et al*, 2008; Dank *et al*, 2008; Koizumi *et al*, 2008; Ajani *et al*, 2009; Kang *et al*, 2009).

The conventional treatment options for advanced gastric patients remain unsatisfactory in terms of response rate, response duration, toxicity, and overall survival benefit. Targeted agents are therefore being investigated in an effort to improve the clinical outcome of gastric cancer patients.

The epidermal growth factor receptor (EGFR) is a transmembrane glycoprotein that is a member of the tyrosine kinase growth factor receptor superfamily. The EGFR represents an important therapeutic target in cancer. The EGFR is expressed in many normal human tissues and has been found to be overexpressed in a large variety of tumours (Ciardiello and Tortora, 2001). In gastric cancer, EGFR is overexpressed in 18–91% of primary tumours and/or metastasis and correlated with a poor prognosis (Tokunaga *et al*, 1995; Albanell *et al*, 2001; Mendelsohn, 2002; Takehana *et al*, 2003; Pinto *et al*, 2007). Cetuximab (Erbitux, Merck KgaA, Darmstadt, Germany) is a human-murine chimeric monoclonal antibody directed to the EGFR binding site. Cetuximab in metastatic colorectal cancer added to standard chemotherapy produces an improvement in the response rate in randomised studies in both a first-line setting and in patients with refractory disease (Cunningham *et al*, 2004; Sobrero *et al*, 2008; Bokemeyer *et al*, 2009; Van Cutsem *et al*, 2009). Cetuximab in combination with chemotherapy regimens containing irinotecan (Pinto *et al*, 2007; Kanzler *et al*, 2009), oxaliplatin (Lordick *et al*, 2007; Han *et al*, 2008, 2009), and cisplatin (Zhang *et al*, 2008; Yeh *et al*, 2009) as first-line therapy of advanced gastric or GEJ adenocarcinoma has also shown promising results.

The docetaxel and cisplatin (DC) combination is an active regimen in advanced gastric cancer patients, and it yielded a response rate of 18.5–26% in two randomised phase II studies (Ajani *et al*, 2005; Roth *et al*, 2007). In preclinical studies, cetuximab enhances the activity of cisplatin (Steiner *et al*, 2007) and docetaxel (Nakata *et al*, 2004). Given these data, this phase II study was performed to evaluate the efficacy and toxicity of a regimen combining a targeted therapy, cetuximab with cisplatin and docetaxel chemotherapy, for unresectable locally advanced or metastatic gastric and GEJ adenocarcinoma.

## Patients and methods

### Study design

We conducted a multicentre phase II study that was approved by the local ethics committee, registered with the health authorities, and performed according to the guidelines of good clinical practice and the Declaration of Helsinki. The primary end point was objective response (ORR). Secondary end points were toxicity, median survival time (OS), and time to progression (TTP).

### Patient and treatment

Patients with advanced, unresectable, histologically confirmed adenocarcinoma of the stomach or GEJ were assessed for eligibility. The eligibility criteria were: age ⩾18 years, Karnofsky performance status (KPS) ⩾70, life expectancy >3 months, no earlier treatment with chemotherapy or radiation therapy, neutrophil count ⩾1500 per *μ*l, platelet count ⩾100.000 per *μ*l, haemoglobin ⩾9.0 g dl^−1^, serum creatinine ⩽1.5 × upper limit of normal (ULN), ALT and AST ⩽2.5 × ULN (⩽5 × ULN in the presence of liver metastases), total bilirubin ⩽1.5 × ULN, measurable disease according to the Response Evaluation Criteria in Solid Tumours (RECIST) (Therasse *et al*, 2000). Prior chemotherapy for advanced cancer was not allowed. Patients who received adjuvant therapy were eligible provided more than 6 months had elapsed between the end of adjuvant therapy and registration for the study. All the patients provided written informed consent for this study, which was approved by the ethics committee of each participating institution. Patients were considered ineligible for the trial if they satisfied any of the following criteria: previous exposure to anti-EGFR, monoclonal antibodies, signal transduction inhibitors, or EGFR targeting therapy; brain metastasis; concurrent malignancy other than non-melanoma skin cancer, or *in situ* cervix carcinoma (patients with an earlier malignancy but with no evidence of disease for >5 years were allowed to enter the trial); clinically relevant coronary artery disease or a history of myocardial infarction within the last 12 months; acute or subacute intestinal occlusion or history of the inflammatory bowel disease; pre-existing neuropathy; known grade 3 or 4 allergic reaction to any of the components of the treatment; pregnancy or lactating status; medical or psychological condition which, in the investigator's opinion, would not enable the patient to complete the study or knowingly sign the informed consent.

The baseline evaluation included a history, physical examination (including evaluation of vital signs and performance status), recording of concomitant medication, laboratory tests (haematology and clinical chemistry, CEA, CA19.9, CA 72.4), thorax and abdomen computed tomography or magnetic resonance imaging, and positron emission tomography scan.

In a collateral study, the KRAS and BRAF mutational status was evaluated. Genomic DNA was extracted from paraffin-embedded primary tumour specimens. The mutational status of KRAS (exon 2) and BRAF (exon 15) was ascertained by PCR amplification followed by direct sequencing.

The phase I study was not performed as no significant increase of chemotherapy toxicity was reported in the earlier phase II studies with cetuximab added to the chemotherapy regimens (Lordick *et al*, 2007; Pinto *et al*, 2007; Han *et al*, 2008; Zhang *et al*, 2008; Han *et al*, 2009; Yeh *et al*, 2009; Kanzler *et al*, 2009).

Patients received cetuximab at an initial dose of 400 mg m^−2^ i.v. followed by weekly doses of 250 mg m^−2^, cisplatin 75 mg m^−2^ i.v. (1-h infusion) plus docetaxel 75 mg m^−2^ i.v. (1-h infusion) on day 1 every 3 weeks, for a maximum of 6 cycles, and then cetuximab alone was allowed in patients with complete response, partial response, and stable disease (maintenance therapy). Cetuximab premedication comprised antihistamine and corticosteroids i.v. All patients received the following supportive medications: 3 l per day hyperhydration; dexamethasone 8 mg (or equivalent drugs) orally administered 12 and 6 h before docetaxel infusion and 8 mg orally twice daily for 4 days after infusion; 5-hydroxytryptamine-3-inhibitors as emesis prophylaxis. In patients benefiting from combination therapy but developing unacceptable intolerance/toxicity to cetuximab or cisplatin/docetaxel, cisplatin/docetaxel or cetuximab may be continued as a single treatment and vice-versa. Surgery of locally advanced gastric cancer could be performed during the study at the time of maximum regression under the earlier assessment of the tumour response after at least 12 weeks of treatment (at least 2 cycles of cisplatin/docetaxel plus cetuximab). If a complete resection was achieved, patients would restart the treatment up to a maximum of 6 treatment cycles (adding pre- and post-surgery treatment).

If a patient experiences grade 3 skin toxicity, cetuximab therapy may be deferred for up to two consecutive infusions without changing the dose level. If the toxicity resolves to grade 2 or less by the following treatment period, the treatment may resume. With the second and third occurrences of grade 3 skin toxicity, cetuximab therapy may again be deferred for up to two consecutive weeks with concomitant dose reductions to 200 and 150 mg m^−2^, respectively. Patients should discontinue cetuximab if more than two consecutive infusions are withheld or a fourth occurrence of grade 3 skin toxicity occurs despite an appropriate dose reduction. Chemotherapy was continued independently of a temporary interruption of cetuximab. Cetuximab was not withheld for cisplatin/docetaxel-related toxicity. Cisplatin/docetaxel dose reduction was planned in the event of severe haematological and/or non-haematological toxicities. Treatment could be delayed for up to 2 weeks to allow toxicities to resolve; delays of >2 weeks required treatment withdrawal. A 15 mg m^−2^ dose reduction of docetaxel or cisplatin was planned for prolonged (>7 days) grade 4 neutropenia, or thrombocytopenia, grade ⩾2 liver toxicity. For grade 2 neurotoxicity, the DC doses were reduced by 15 mg m^−2^. Cisplatin was reduced or discontinued if creatinine clearance was <60 or <40 ml min^−1^. Treatment was discontinued for persistent grade 3 liver toxicity, anaphylaxis, or fluid retention; grade ⩾3 neuropathy; or a recurrent toxicity despite dose reductions.

### Response and toxicity

Routine evaluation of patients was performed on a weekly basis during therapy. These evaluations included a physical examination, vital signs, KPS, laboratory haematological and serum chemistry, and the recording of adverse events.

The evaluation tumour response to therapy was based on a computed tomography or magnetic resonance scan. Patients were evaluable for response if they had received at least one course of therapy. In addition, those patients who were developing rapid tumour progression, or those who died of progressive disease before the response evaluation, would also be considered evaluable for response. Also, patients who discontinued treatment, or those who died from a treatment-related toxicity before response evaluation, were considered evaluable for response. The tumour was evaluated every 6 weeks during the treatment and at least every 12 weeks during the follow-up. The RECIST criteria were used to assess the type of response (Therasse *et al*, 2000). Positron emission tomography scan was performed after 3 weeks, before the second cycle of cisplatin/docetaxel chemotherapy. Toxicity was graded according to the National Cancer Institute Common Toxicity Criteria (CTC) Version 3.0 (National Cancer Institute, Common Toxicity Criteria, 2006).

### Statistical analysis

The statistical design was performed according to Simon's two-stage design (Simon, 1989). It was expected that the new regimen would have a targeted response rate ⩾40%. The first stage required at least six or more patients out of 20 to have a confirmed partial or complete response assuming P1=0.40, P0=0.25, with *α*=0.05 and *β*=0.2 before proceeding to the second stage. In the second stage, 40 assessable patients could be added and if a total of 24 or more patients achieved a confirmed objective response, then the primary end point would have been met. Seventy-two patients were thus needed to take account of possible attrition. TTP and OS were calculated using the Kaplan–Meier method (Kaplan and Meier, 1958). Descriptive statistics were used for safety evaluation.

## Results

### Patient population

From December 2007 to August 2008, 72 patients, in five Italian centres, were enrolled. All 72 patients were evaluated for safety and OS calculations, and 68 were assessable for response. Four patients were not assessable for ORR because one patient with peritoneal carcinomatosis died as a result of bowel occlusion in week 5 of treatment; one died of gastric bleeding in week 1; one died of bowel perforation in week 3; and 1 left the study because of renal toxicity grade 3 after the first chemotherapy cycle.

The patient characteristics are listed in Table 1. The majority of patients were males (66.7%), their median age being 63 years (range, 18–75) and the median KPS 90 (range, 70–100). GEJ was involved in 13 patients (18.1%). The histotype was intestinal adenocarcinoma in 41 patients (56.9%) and non-intestinal adenocarcinoma in 31 (43.1%). At the baseline of the study, three patients (4.2%) had non-resectable locally advanced disease and 69 (95.8%) had metastatic disease. The majority of patients (76.4%) had two or more metastases sites. Nine patients (12.5%) had received prior adjuvant chemotherapy. Four patients had adjuvant chemotherapy containing cisplatin.

### Treatment administration

Altogether, 1049 weeks of cetuximab were administered, with a median of 13.5 weeks (range, 1–35), and 325 cycles of cisplatin/docetaxel, with a median of 5 cycles (range, 1–6). The median relative dose intensity was 1.0 for cetuximab (range, 0.1–1.0), 1.0 for cisplatin (range, 0.5–1.0), and 1.0 for docetaxel (range, 0.3–1.0) (Table 2).

Cetuximab was discontinued in two patients (2.8%) for grade 3 infusion-related reaction during the first administration; no cetuximab reduction dose was performed. One patient stopped docetaxel administration for grade 3 hypersensitivity reaction during the first cycle. At least a one-dose reduction of cisplatin was required in 20 patients (27.8%) and of docetaxel in 19 patients (26.4%). Neutropenia was the most frequent reason for dose reduction or delay.

Maintenance cetuximab therapy (after 6 cycles of cisplatin/docetaxel plus cetuximab treatment) was performed in 16 (51.6%) out of 31 eligible patients (31.6%). The median duration of maintenance treatment was 10 weeks (range, 2–17).

### Response

The data on the treatment response are listed in Table 3. One patient (1.5%) achieved a complete response and 27 patients (39.7%) achieved a partial response; the ORR was 41.2% (95% CI, 29.5–52.9). Twenty-four patients (33.3%) had stable disease and 16 patients (23.5%) had progressive disease. The disease control rate (complete response plus partial response plus stable disease) was 76.5%. The median time to response was 6 weeks (range, 6–19). The median duration of response was 5 months (range, 1–22 months).

The objective response was similar in both histotypes (45.9% for intestinal adenocarcinoma and 35.5% in non-intestinal adenocarcinoma; *P*=0.27). The ORR in patients with grade ⩾2 skin reactions after cetuximab therapy was higher, but not statistically significantly, than the ORR in patients with <2 grade (*P*=0.05) (Table 4).

Two responding patients with non-resectable locally advanced disease were submitted to surgery. One patient achieved a partial response after 4 cycles of cisplatin/docetaxel plus cetuximab therapy and after gastrectomy R0 received the other 2 cycles of combination therapy and 12 weeks of maintenance cetuximab (patient alive and disease-free after 26 months from the start of treatment); and one submitted to gastrectomy R0 after 6 cycles of combination therapy (patient alive and disease-free after 9 months from the start of treatment). Another responder patient with peritoneal metastases who submitted to gastrectomy after 6 therapy cycles has achieved a pathologic complete response (patient alive with disease relapse 16 months after the start of treatment).

Twenty-five (34.7%) patients received a second-line treatment: 13 with 5-fluorouracil, folinic acid and irinotecan (FOLFIRI), 5 with etoposide, folinic acid, and 5-fluorouracil (ELF), 3 with capecitabine, 3 with 5-fluorouracil, and 1 with epirubicin and 5-fluorouracil.

### Time-to-progression and survival

**Figure 1:**
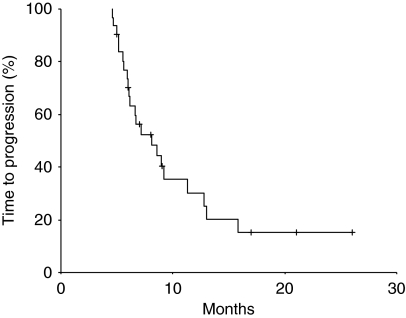
Time to progression.

**Figure 2:**
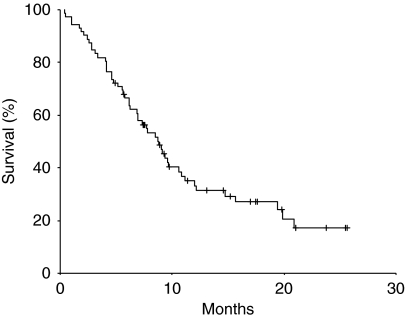
Overall survival.

The median follow-up time was 19 months (range, 6–26 months). The median TTP was 5 months (95% CI, 3.7–5.4) (Figure 1). The median OS time was 9 months (95% CI, 7–11) (Figure 2). In the setting of patients with at least stable disease after 6 cycles of cisplatin/docetaxel plus cetuximab eligible for cetuximab maintenance treatment, patients treated with maintenance cetuximab (No.=16) compared with no-maintenance treatment (No.=15) showed a trend for longer TTP of 9.2 months (95% CI, 8.1–10.1) *vs* 6.6 (95% CI, 5.3–6.7) (*P*=0.10), and OS of 19.8 months (95% CI, 12.3–27.4) *vs* 7.7 (95% CI: 5.1–10.3) (*P*=0.22).

### Safety

All 72 patients were evaluated for toxicity (Table 5). The major toxicity observed was haematological. Grades 3–4 neutropenia occurred in 32 patients (44.4%). Febrile neutropenia was recorded in 14 (19.4%) patients. Overall, non-haematological toxicities were moderate, and severe episodes were rare. The most common grades 3–4 non-haematological toxicities were asthenia (16.7%), hypokalemia (12.5%), vomiting (8.3%), stomatitis (4.2%), and diarrhoea (4.2%). Cetuximab-related hypersensivity reaction was reported in 2 patients (2.8%). All grades of acne-like rash occurred in 51 patients (70.8%), and grades 3–4 was observed in 12 (16.7%). Grades 1–4 of hypomagnesemia was recorded in 20 of 32 patients with available data (62.5%), and grades 3–4 was observed in only 1 patient (3.1%).

Eight deaths occurred within 60 days from the start of therapy: one due to bowel occlusion, one due to bowel perforation, one due to gastric bleeding, and five due to progression of disease. No toxic death was observed.

### Collateral studies

KRAS and BRAF mutational status was evaluated in 32 of 72 patients (44.4%). *KRAS* mutations were detected in 3 (9.4%) of the tumours analysed. Two cases displayed amino acid substitutions of codon 12 and 1 of codon 13. No *BRAF* mutations were found. In this patient cohort, oncogenic activation of *KRAS* was not significantly associated with the objective response.

The data of early 18F-FDG-PET assessment will be reported in a subsequent publication.

## Discussion

Unresectable advanced or metastatic gastric cancer still has a poor prognosis, with a median survival of <12 months. A recent meta-analysis has confirmed that 5-fluorouracil-based regimens provide superior survival in patients with advanced gastric cancer as compared with those treated with the best supportive care (HR, 0.39; 95% CI, 0.28–0.52). This meta-analysis found a statistically significant advantage in survival (*P*=0.001) in favour of combination chemotherapy compared with a single agent (HR, 0.83; 95% CI, 0.74–0.93). In addition, combination chemotherapy regimens containing 5-fluorouracil, anthracycline, and cisplatin were associated with a significant survival benefit when compared with 5-fluorouracil and anthracycline regimens without cisplatin (HR, 0.83; 95% CI, 0.76–0.91) (Wagner *et al*, 2006).

Recently developed new agents, such as docetaxel, irinotecan, oxaliplatin, capecitabine, and S-1, have been investigated in phase III clinical trials (Van Cutsem *et al*, 2006; Cunningham *et al*, 2008; Dank *et al*, 2008; Koizumi *et al*, 2008; Ajani *et al*, 2009; Kang *et al*, 2009). Although some combinations, such as epirubicin, oxaliplatin, and capecitabine (EOX) in a REAL 2 study, have been shown to be an interesting as well as effective regimen (overall survival 11.2 months; HR, 0.80; 95% CI, 0.66–0.97; *P*=0.02, compared con ECF) (Cunningham *et al*, 2008), no worldwide standard regimens have been established.

In the three-arm SAKK42/99 phase II study, the patients as first-line treatment of advanced gastric cancer were randomised to receive the DC regimen (docetaxel at a dose of 85 mg m^−2^ on day 1 and cisplatin at a dose of 75 mg m^−2^ on day 1), or the DCF regimen (docetaxel at a dose of 75 mg m^−2^, cisplatin at a dose of 75 mg m^−2^ on day 1, and 5-fluorouracil at a dose of 300 mg m^−2^ per day as a continuous infusion on days 1–14), or the ECF regimen (epirubicin at a dose of 50 mg m^−2^, cisplatin at a dose of 60 mg m^−2^ on day 1, and 5-fluorouracil at a dose of 200 mg m^−2^ per day as a continuous infusion on days 1–21) every 3 weeks. In the preliminary results on 119 patients, the ORR, TTP, and OS of DC *vs* DCF *vs* ECF were 18.5 *vs* 36.6 *vs* 25%, 4.4 *vs* 7.8 *vs* 5.4 months, and 11 *vs* 10.4 *vs* 8.2 months, respectively. Grades 3–4 neutropenia was observed in 49% on the DC arm, 57% on the TCF arm, and 34% on the ECF arm. Gastrointestinal grades 3–4 toxicities (diarrhoea, stomatitis) were observed in 3% on the DC arm, 22% on the TCF arm, and 11% on the ECF arm (Roth *et al*, 2007).

In phase II of the V-325 study, first-line DC (docetaxel at a dose of 85 mg m^−2^ on day 1 and cisplatin at a dose of 75 mg m^−2^ on day 1) and DCF (docetaxel at a dose of 75 mg m^−2^, cisplatin at a dose of 75 mg m^−2^ on day 1, and 5-fluorouracil at a dose of 750 mg m^−2^ per day as a continuous infusion on days 1–5), both administered every 3 weeks, were compared in 158 patients with advanced gastric or GEJ adenocarcinoma. Both regimens were active. ORR, TTP, and OS of DC *vs* DCF were 26 *vs* 43%, 5.0 *vs* 5.9 months, and 10.5 *vs* 9.6 months, respectively. The most frequent grades 3–4 adverse events were neutropenia (87% in the DC group *vs* 86% in the DCF group) and gastrointestinal toxicities (30% in the DC group *vs* 56% in the DCF group) (Ajani *et al*, 2005). In phase III of the V-325 trial, a total of 445 patients were randomised and treated with DCF every 3 weeks or with CF (cisplatin at a dose of 100 mg m^−2^ on day 1, and 5-fluorouracil at a dose of 1000 mg m^−2^ per day as a continuous infusion on days 1–5), every 4 weeks. All efficacy end points, TTP (primary end point), OS, and ORR, were found to be significantly improved in the DCF arm *vs* the CF arm 5.6 *vs* 3.7 months, (HR, 1.47; 95% CI, 1.19–1.82; *P*<0.001), 9.2 *vs* 8.6 months (HR, 1.29; 95% CI, 1.0–1.6; *P*=0.02), and 37 *vs* 25% (*P*=0.01), respectively. DCF was associated with increased toxicity, compared with CF, especially grades 3–4 neutropenia (82.3 *vs* 56.8%), and febrile neutropenia (30 *vs* 13.5%) (Van Cutsem *et al*, 2006). Following the results of V-325, several variations of DCF have been reported with different administration schedules, alternative platinum and fluoropyrimidine components to try to optimise both efficacy and safety (Park *et al*, 2005; Orditura *et al*, 2006; Lorenzen *et al*, 2007; Tebbutt *et al*, 2007).

Molecular targeting agents are another new topic in the field of cancer therapy, and may provide a significant impact also in gastric cancer treatment, as successful results have been observed in colorectal cancer. An increase of EGFR protein expression in gastric cancer appears to be related to biological aggressiveness, although gene amplification has occurred only to a small extent (Tokunaga *et al*, 1995; Albanell *et al*, 2001; Mendelsohn, 2002; Takehana *et al*, 2003). Numerous studies have established that gastric cancer has overexpressed many growth factors and their receptors, including the EGF family, and numerous cytokines, such as transforming growth factor (Zheng *et al*, 2004). The presence of EGF in gastric cancer is correlated with the degree of gastric wall invasion and lymph node metastasis. The 5-year survival of patients with EGF-positive tumours is worse than that of patients with EGF-negative tumours (Albanell *et al*, 2001; Mendelsohn, 2002; Takehana *et al*, 2003). A recent study has shown that hereditary diffuse gastric cancer-associated E-cadherin germline missense mutations lead to increased EGFR activity (Moutinho *et al*, 2008). Several phase II trials have reported on the promising clinical activity of cetuximab in combination with chemotherapy. The chemotherapy regimens included irinotecan (FOLFIRI, FUFIRI), oxaliplatin (FUFOX, FOLFOX, XELOX), and cisplatin (capecitabine/cisplatin). All chemotherapy regimens contained a fluoropyrimidine. The ORR varied from 40 to 67% in these phase II studies (Pinto *et al*, 2007; Lordick *et al*, 2007; Han *et al*, 2008; Zhang *et al*, 2008; Han *et al*, 2009; Yeh *et al*, 2009; Kanzler *et al*, 2009).

In the DOCETUX study, the addition of cetuximab to the DC regimen (cisplatin and docetaxel at a dose of 75 mg m^−2^ for both drugs) was associated with an improvement in the ORR compared with the results obtained by the regimen DC alone in randomised phase II studies. The ORR was 41.2% of DC plus cetuximab in the DOCETUX study, and 18.5 and 26%, respectively, of DC alone in the SAKK42/99 and V-325 studies. The DC plus cetuximab treatment achieved the same ORR in both different histotypes, intestinal and non-intestinal adenocarcinoma. We observed a trend (not statistically significant) between treatment activity and the severity of cetuximab-induced skin reactions, as reported in the other study with cetuximab-based therapy (Cunningham *et al*, 2004; Jonker *et al*, 2007; Pinto *et al*, 2007; Bokemeyer *et al*, 2009). An additional finding was that the DC plus cetuximab regimen resulted in a short time to response (median 6 weeks). This suggests that this combination may also be a potentially effective treatment in a neoadjuvant setting, where a rapid tumour reduction before curative surgery is important. Three patients in the DOCETUX trial (two with locally advanced disease and 1 with peritoneal metastases) have obtained remission after treatment that has permitted radical surgery.

In this study, we have a marginal increase in TTP (5 months) and lack of improvement in OS (9 months). These OS data may be the result of the unfavourable characteristics of the patients: 43.1% with non-intestinal histotype (77.4% with signet ring cells), 95.8% with metastatic disease, and 76.4% with ⩾2 organs involved. Furthermore, the median age of the patients was 63 years and 39% of the patients were ⩾65 years old (higher than the V-325 study with a median age of 55 years and 24% of patients ⩾65 years old (Van Cutsem *et al*, 2006). Although the analysis was not planned, this study suggests a non-statistically significantly favourable disease control trend in patients treated with cetuximab maintenance compared with patients without maintenance treatment (TTP 9.2 *vs* 6.6 months; OS 19.8 *vs* 7.7 months). The possibly favourable impact on TTP and OS of cetuximab maintenance treatment in patients who had at least stable disease after chemotherapy plus cetuximab therapy was also suggested by the results of our earlier phase II study in advanced gastric cancer patients treated with FOLFIRI plus cetuximab (FOLCETUX Study) (Pinto *et al*, 2007) and phase III study in recurrent or metastatic head and neck cancer patients treated with platinum-based chemotherapy plus cetuximab (EXSTREME Study) (Vermorken *et al*, 2008).

The major toxicity of the DOCETUX treatment appears to be limited to neutropenia (44.4% of grades 3–4, with 19.4% of febrile neutropenia). Gastrointestinal toxicities (stomatitis, diarrhoea) were modest (8.4%). Overall, the side effects were moderate. The toxicity of DC chemotherapy was unaffected by the addition of cetuximab. The percentage of grades 3–4 neutropenia in the DOCETUX regimen was lower that in the DC regimen of the SAKK42/99 study (76% of grades 3–4, with 21% of febrile neutropenia) and V-325 study (87% grades 3–4, with 27% of febrile neutropenia). These data could be related to a lower dose of cisplatin (75 mg m^−2^) in the DOCETUX study. The specific side effects associated with cetuximab were skin reactions (70.8% all grades, and 16.7% of grades 3–4), infusion-related reactions (2.8% of grade 3), and hypomagnesemia (62.5% all grades, and 1.4% of grades 3–4). Compliance with cetuximab was good, with only two patients (2.8%) discontinuing the treatment for grade 3 infusion-related reactions. There were no treatment-related deaths.

In the DOCETUX phase II study, cetuximab in combination with the cisplatin/docetaxel regimen increases the ORR of the cisplatin/docetaxel doublet and allows for a well-tolerated dose of both drugs. The lack of improvement in OS could be correlated to the unfavourable characteristics of the patients, such as the absence of a fluopyrimidine in the chemotherapy regimen. These results support new trials in the first-line regimen of advanced gastric or GEJ adenocarcinoma. Future studies should investigate the major active chemotherapy triplet with schedules of 5-fluorouracil (or oral fluopyrimidines) plus cisplatin and docetaxel in combination with cetuximab under different settings.

## Figures and Tables

**Table 1:** Patient characteristics

	**No. 72**	
**Characteristics**	**No.**	**%**
*Sex*		
Male	48	66.7
Female	24	33.3
		
*Age, years*		
Median	63	
Range	18–75	
		
*Karnofsky performance status*		
100	31	43.1
90	18	25
80	18	25
70	5	6.9
		
*Primary tumour site*		
Stomach	59	81.9
Gastro-oesophageal junction	13	18.1
		
*Histotype*		
Intestinal adenocarcinoma	41	59.9
Non-intestinal adenocarcinoma^a^	31	43.1
		
*State of disease*		
Locally advanced	3	4.2
Metastatic	69	95.8
		
*Prior surgery*		
Total/partial gastrectomy	25	34.7
Protesis/anastomosis	7	9.7
		
*Adjuvant chemotherapy*	9	12.5
5-FU/AF regimens	5	55.6
5-FU/CDDP regimens	2	22.2
5-FU/CDDP/EPI regimens	2	22.2
		
*Site of disease*		
Primary	47	65.3
Lymph nodes	47	65.3
Liver	35	48.6
Peritoneum/local recurrence	26	36.1
Other	14	19.4
		
*No. of organs involved*		
1	17	23.6
2	27	37.5
>2	28	38.9

a 24 (77.4%) with signet ring cells.

**Table 2:** Delivery of treatment

	**Cetuximab**	**Cisplatin**	**Docetaxel**
No. of drug administration	1049 (weeks)	325 (cycles)	
Median of weeks/cycles	13.5	5	
Range	1–35	1–6	
Median relative dose intensity	1.0	1.0	1.0
Range	0.1–1.0	0.5–1.0	0.3–1.0
Dose reduction, no. (%)	1 (1.4)	20 (27.8)	19 (26.4)
Drug discontinuation, no. (%)			
Temporary	19 (26.4)	8 (11.1)	6 (8.3)
Definitive	2 (2.7)	0	1 (1.4)

**Table 3:** Response rate

	**No. 68**	
**Response**	**No.**	**%**
Complete response	1	1.5
Partial response	27	39.7
Overall response rate	21	41.2
(95% CI)	(29.5–52.9)	
Stable disease	24	33.3
Progressive disease	16	23.5
Disease control rate	52	76.5

**Table 4:** Response rate according to histotype and skin rash

	**Intestinal (no. 37)**		**Non-intestinal (no. 31)**			**Skin rash <2 (no. 36)**		**Skin rash ⩾2 (no. 32)**		
**Response**	**No.**	**%**	**No.**	**%**		**No.**	**%**	**No.**	**%**	
CR	1	2.7	0	—		0	—	1	3.1	
PR	16	43.2	11	35.5		11	30.6	16	50	
ORR (95% CI)	17	45.9 (33.1–58.7)	11	35.5 (13.6–35.7)	*P*=0.27	11	30.6 (10.7–31.8)	17	53.1 (24.5–49.3)	*P*=0.05
SD	14	37.8	10	32.3		11	30.6	13	20.3	
PD	6	16.2	10	32.3		14	38.9	2	6.2	

**Table 5:** Toxicity

	**No. 72**			
	**All grades**		**Grades 3–4**	
**Toxicity**	**No.**	**%**	**No.**	**%**
*Haematological toxicity*				
Neutropenia	41	56.9	32	44.4
Febrile neutropenia	NA	—	14	19.4
Anemia	29	40.3	4	5.6
Thrombocytopenia	13	18.1	2	2.8
				
*Non-haematological toxicity*				
Acne-like rash	51	70.8	12	16.7
Diarrhea	23	31.9	3	4.2
Asthenia	45	62.5	12	16.7
Stomatitis	26	36.1	3	4.2
Hypertransaminasemia	11	15.3	0	—
Hyperbilirubinemia	10	13.9	3	4.2
Vomiting	22	30.6	6	8.3
Anorexia	28	38.9	2	2.8
Nausea	35	48.6	8	11.1
Alopecia	33	45.8	NA	—
Hypoacusia	1	4.2	0	—
Neurotoxicity	4	5.6	1	1.4
Hypomagnesemia^a^	20	62.5	1	3.1
Hyponatremia^a^	14	43.8	8	25
Hypokalemia^a^	11	34.4	4	12.5

NA=not applicable.

a Data available in 32 patients.
